# Research progress on the impact of curcumin on immune responses in breast cancer

**DOI:** 10.1515/biol-2025-1089

**Published:** 2025-09-08

**Authors:** Xiao Han, Renzhe Tang, Peng Wang, Li Liu

**Affiliations:** Department of Oncology, First Affiliated Hospital, Heilongjiang University of Chinese Medicine, Harbin 150000, Heilongjiang, China; The First Department of Cardiovascular, First Affiliated Hospital, Heilongjiang University of Chinese Medicine, Harbin 150000, Heilongjiang, China

**Keywords:** curcumin, breast cancer, immune response, cell apoptosis, therapeutic approaches

## Abstract

The Latin scientific name of turmeric is *Curcuma longa* L., and it belongs to the Zingiberaceae plant family. Curcumin is a yellow compound extracted from the rhizomes of turmeric, known for its various biological activities, including antioxidant, anti-inflammatory, and anticancer properties. This study presents a comprehensive review of the relationship between curcumin and the immune response in breast cancer (BC). Specific therapeutic approaches of curcumin for BC treatment are summarized. The anti-tumor activity of curcumin has garnered significant attention, with unique immunomodulatory effects on inhibiting cancer cell proliferation, inducing autophagy, affecting the cell cycle, and regulating cell apoptosis. Curcumin enhances immune cell-mediated actions against cancer cells through modulation of immune response pathways, alteration of the tumor microenvironment, and influencing immune cell function. Curcumin, via multiple pathways such as anti-inflammatory, antioxidant, apoptosis-inducing, and immunomodulatory effects, holds important clinical value in BC therapy.

## Graphical abstract

**Figure:**
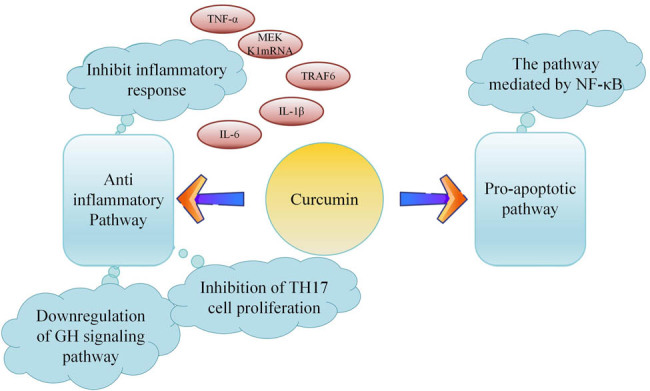

## 1 Introduction

Breast cancer (BC) is the most prevalent malignant tumor among women worldwide. In 2022, the global incidence and mortality of BC in women were approximately 2.309 million and 666,000 cases, ranking second and fourth, respectively, among all malignancies [1]. It is projected that by 2040, the incidence and mortality rates of BC will increase by 40 and 50%, respectively, due to population growth and aging [2]. Clinically, BC presents several subtypes, including hormone receptor-positive, human epidermal growth factor receptor 2 (HER2) overexpression, and triple-negative subtypes [3]. These subtypes exhibit distinct biological behaviors and treatment sensitivities, thereby necessitating personalized therapeutic approaches. In addition, BC presents heterogeneity in clinical features, including tumor size, lymph node involvement, and histological grading. This complexity and diversity underscore the need for a comprehensive approach in BC clinical management, considering multiple factors to develop optimal treatment strategies. Traditional treatment modalities for BC include surgical resection, radiotherapy, chemotherapy, and endocrine therapy. Although these approaches have achieved some success, limitations persist, including poor prognosis, adverse effects, and drug resistance [4].

In the search for new therapeutic strategies, scientists have increasingly turned their attention to natural products, particularly those compounds with anti-inflammatory, antioxidant, anti-proliferative, and immune-modulatory properties [5]. As part of complementary and alternative medicine, plant-based therapies have been extensively researched and applied over the past few decades. These therapies are often grounded in centuries-old traditional medical knowledge, with several of them being validated by modern science as effective adjuncts in cancer treatment. For instance, curcumin, a polyphenolic compound extracted from the rhizome of turmeric, has garnered significant attention due to its distinctive biological activities.

The history of curcumin dates back thousands of years in traditional Asian medicine, such as Ayurveda and Traditional Chinese Medicine, where it was used as a spice, dye, and treatment for various health conditions [6]. In recent years, an increasing body of research has shown that curcumin not only possesses potent anti-inflammatory and antioxidant properties but also exhibits significant anti-tumor effects against various types of cancer [7]. Its mechanisms of action are multifaceted, involving the inhibition of cancer cell proliferation, induction of apoptosis, alteration of the cell cycle, modulation of tumor-associated signaling pathways, and enhancement of the immune system to combat cancer. Studies demonstrated that curcumin can promote apoptosis in BC cells and influence their cell cycle progression [8]. Furthermore, research indicates that curcumin effectively reduces the viability of human pancreatic cancer cell lines, with IC_50_ values ranging from 8.67 to 20.35 µmol/L, showing strong anti-proliferative activity [9].

However, the immune response in BC is a complex process that involves the participation of various immune cells, including T cells, B cells, macrophages, and natural killer (NK) cells. An ideal immune response should eliminate cancer cells, suppress tumor growth and metastasis, and thereby control or cure the disease. Unfortunately, tumors can evade immune surveillance through mechanisms such as altering antigen expression, inhibiting immune cell activity, or inducing immune tolerance, which limits the effectiveness of the immune response. Therefore, the research and development of novel therapeutic strategies that can enhance the immune response are crucial for improving the efficacy of BC treatment.

Given the unique properties of curcumin and its impact on the immune response in BC, this review focused on how curcumin modulated the immune system to combat BC, evaluating its potential as a therapeutic option. This work discussed in detail the pharmacological effects of curcumin, its specific impact on the BC immune response, and the latest research developments in this area. It was hoped that this review would not only provide valuable insights into curcumin and its application in BC treatment but also inspire further exploration of plant-based therapies and natural products in cancer treatment.

## 2 Characteristics of curcumin

**Figure 1:**
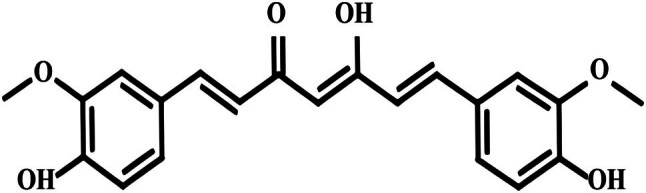
Structural diagram of curcumin.

Curcumin is primarily extracted from the rhizomes of Zingiberaceae plants such as turmeric (*Curcuma longa* L.) and zedoary (*Curcuma zedoaria* (Berg.) Rosc.), and additionally, the rhizomes of plants from the Araceae family, such as calamus (*Acorus calamus* L.), serve as another important source of curcumin [10]. Its chemical formula is C_21_H_20_O_6_ (Figure 1). Its earliest recorded medicinal use dates to the 6th century BCE in ancient Indian medical texts. For a long period thereafter, curcumin was primarily used as a food additive. It was not until 1910 that researchers deduced the accurate chemical structure of curcumin, leading to subsequent systematic investigations into its chemical properties and pharmacological effects [11]. Curcumin is a polyphenolic compound characterized by two benzene rings and an elongated side chain in its chemical structure. It possesses potent antioxidant and anti-inflammatory properties, mitigating oxidative damage and inflammatory responses by interacting with free radicals. Research has indicated that curcumin exhibits antioxidative, anti-inflammatory, anti-tumor, anti-platelet aggregation, antibacterial, antiviral, and cholesterol-lowering effects. Additionally, some studies have suggested its neuroprotective and cardioprotective properties. Curcumin also holds potential benefits in the prevention and treatment of diseases such as cancer, cardiovascular disorders, diabetes, and arthritis [12].

In 1985, pioneering research first proposed the anticancer properties of curcumin, highlighting its potential in counteracting inflammation and preventing cancer by scavenging free radicals or activating endogenous mechanisms to counteract oxidative stress triggered by carcinogenic agents. Subsequent studies indicated that curcumin not only exerts a direct inhibitory effect on tumor cell growth but also modulates multiple signaling pathways and molecular targets, thereby inhibiting processes such as tumor metastasis, invasion, and angiogenesis [13]. These findings have propelled further exploration and investigation of curcumin as a potential anticancer agent.

Although curcumin is renowned for its potent antioxidant and anti-inflammatory properties, which alleviate oxidative damage and inflammatory responses through interaction with free radicals, its application in BC treatment faces several key challenges. First, curcumin has low bioavailability, primarily due to its poor water solubility and rapid metabolism. This low bioavailability limits curcumin’s ability to reach effective concentrations *in vivo*, thereby affecting its clinical efficacy. Second, studies noted that curcumin can induce apoptosis in cancer cells and alter the cell cycle; however, this effect may vary across different BC subtypes, particularly in tumors with strong drug resistance [14].

Curcumin, a natural compound with potential anticancer activity, faces limitations in its application due to its low bioavailability. To overcome this challenge, scientists have explored various delivery strategies, particularly the use of nanotechnology and liposomal technology. Polymer-based nanoparticles have attracted attention due to their good biocompatibility and adjustable release characteristics. For instance, poly(lactic-co-glycolic acid) (PLGA) nanoparticles have been extensively studied to improve drug stability and prolong its therapeutic duration. In contrast, lipid-based nanoparticles, such as solid lipid nanoparticles and nanostructured lipid carriers, are renowned for their high drug-loading capacity and better physical stability. Inorganic nanoparticles, such as silica or gold nanoparticles, although posing challenges in terms of biodegradability, offer unique optical and electrical properties, making them suitable for imaging-guided therapy. By comparing the advantages and disadvantages of these different nanoparticle systems, a basis can be established for selecting the most suitable carrier for curcumin delivery.

Additionally, liposomes, as another important form of drug delivery system, are directly influenced by the selection of their components, which affects encapsulation efficiency, stability, and drug release behavior. Common components, such as phosphatidylcholine (PC) and cholesterol, are used to construct stable liposomal structures. Increasing the proportion of cholesterol can enhance the stability of liposomes and reduce drug leakage. Furthermore, surface modification techniques, such as PEGylation, can improve the circulation time and targeting ability of liposomes. By thoroughly evaluating the combinations of different liposomal components and their stability characteristics, the delivery efficacy of curcumin can be optimized.

In addition to traditional nanoparticles and liposomes, novel delivery systems such as dendrimers and hydrogels have shown great potential. Dendrimers, with their highly branched three-dimensional structure, are capable of efficiently encapsulating and protecting curcumin while providing high cellular uptake efficiency. Hydrogels, owing to their excellent biocompatibility and easily tunable physicochemical properties, demonstrate advantages in local drug delivery. These novel delivery systems not only enhance the solubility and stability of curcumin but also improve its targeting specificity and reduce side effects.

The efficacy of each delivery system needs to be further validated through detailed pharmacokinetic studies. Data on the absorption, distribution, metabolism, and excretion (ADME) processes of the drug *in vivo* can help us better understand how different formulations affect the bioavailability of curcumin. For example, nanoparticles may extend the drug’s half-life by altering its tissue distribution pattern, while liposomes could improve bioavailability by reducing the hepatic first-pass effect.

Finally, although laboratory studies demonstrated the potential of these delivery systems in enhancing curcumin’s bioavailability, many challenges remain in the clinical translation process. Current research is largely limited to animal experiments, and further clinical trials are needed to verify their safety and efficacy. Additionally, cost-effectiveness analysis is an important consideration, especially in large-scale production.

The dose-dependent toxicity of curcumin has been explored in several preclinical and clinical studies. In animal models, high doses of curcumin may lead to side effects such as gastrointestinal discomfort, elevated liver enzyme levels, and immune system suppression [15]. However, in human studies, curcumin generally demonstrates good tolerance, with no severe toxic reactions observed even at higher doses [16]. Nonetheless, further research is needed to determine the optimal dosage range in different populations and to clarify its dose-response relationship.

When used in combination with various chemotherapeutic agents, curcumin may produce either synergistic or antagonistic effects. For instance, studies have shown that curcumin can enhance the anticancer effects of cisplatin while reducing its nephrotoxicity. On the other hand, curcumin may also affect the metabolic pathways of certain drugs, particularly by inhibiting the cytochrome P450 enzyme system, which can increase the plasma concentrations and potential toxicity of other drugs. Therefore, careful consideration of the interactions between curcumin and other chemotherapeutic agents is essential when designing combination therapies to avoid unnecessary side effects and optimize therapeutic outcomes.

The long-term safety of curcumin is another important consideration. Although short-term use of curcumin is generally considered safe, data on its long-term safety are relatively limited. Overall, the incidence of adverse events associated with curcumin is low, but further large-scale clinical trials are needed to validate its safety. Safety data for pregnant women, lactating women, and children are particularly limited. Given that curcumin may impact fetal development or breastfeeding, it should be used with caution in these populations. There may be individual variations in response to curcumin, potentially linked to genetic factors. Future research should focus on the impact of genetic polymorphisms on curcumin metabolism and efficacy to inform personalized treatment strategies. As a natural product, curcumin is often used in combination with other herbs or supplements. Understanding how these combinations affect curcumin’s bioavailability and safety is crucial. In conclusion, while existing evidence suggests that curcumin has good safety, a comprehensive safety assessment is still needed before its widespread use in BC treatment.

## 3 Impact of curcumin on the immune response in BC

Given the inherent challenges posed by tumor immune evasion mechanisms, immune cell functional states, and immune suppression within the tumor microenvironment (TME), curcumin’s multifaceted effects – such as its antioxidant, anti-inflammatory, anti-tumor, anti-platelet aggregation, antimicrobial, antiviral, and cholesterol-lowering properties – regulate the immune response by promoting anti-tumor T-cell activity and altering the cytokine milieu. These effects may address issues observed in BC immune responses, potentially leading to improvements in BC immunotherapy.

Curcumin has been shown to alter macrophage polarization, converting them from the pro-tumor M2 phenotype to the anti-tumor M1 phenotype [17]. This transition helps create a microenvironment conducive to immune-mediated tumor cell clearance. Additionally, curcumin can enhance the activity of NK cells, increasing their ability to recognize and kill tumor cells. Curcumin interferes with key signaling pathways, such as the phosphoinositide 3-kinase (PI3K)/AKT serine (AKT)/threonine kinase/mammalian target of rapamycin pathway, which are closely associated with tumor cell survival. By inhibiting these pathways, curcumin not only reduces cancer cell proliferation but also promotes apoptosis [18]. Moreover, curcumin upregulates p53 gene expression, further enhancing its anti-cancer effects. In recent years, immune checkpoint inhibitors have become one of the effective strategies for treating certain types of cancer. Curcumin has shown synergistic effects with these drugs, such as enhancing T-cell-mediated anti-tumor immune responses by reducing PD-L1 expression levels.

The combined mechanism of action of curcumin and immune checkpoint inhibitors involves multiple levels. First, curcumin can inhibit the inflammatory response by modulating the nuclear factor kappa-light-chain-enhancer of activated B cells (NF-κB) signaling pathway and reduce the expression of PD-L1, thereby relieving the immune suppression within the TME. Second, curcumin can activate the Nrf2 pathway, enhancing the antioxidant defense system and protecting immune cells from oxidative stress-induced damage, thus maintaining their functional integrity. Furthermore, curcumin promotes the maturation and antigen-presenting capacity of dendritic cells, further enhancing T-cell-mediated anti-tumor immune responses. This multi-target, multi-level mechanism of action makes curcumin an ideal candidate for combination therapy with immune checkpoint inhibitors.

Currently, clinical trials on the combination of curcumin and immune checkpoint inhibitors are still in the early stages. Although some preliminary studies suggest that this combination holds potential, most research is focused on preclinical models or small-scale Phase I/II clinical trials. The combination of curcumin and immune checkpoint inhibitors may yield significant anti-tumor effects. In terms of safety, while curcumin is generally considered safe, high doses may cause mild-to-moderate gastrointestinal discomfort and other side effects. Therefore, optimizing the dosage and administration regimen of curcumin is crucial for ensuring the safety of combination therapy.

Determining the optimal sequencing strategy is essential for maximizing the efficacy of the combination therapy of curcumin and immune checkpoint inhibitors. Studies have shown that different dosing sequences may affect treatment outcomes. For example, administering curcumin first to prepare the TME, followed by immune checkpoint inhibitors, may be more effective than simultaneous administration. Additionally, developing personalized treatment plans based on the patient’s specific condition and biomarker characteristics is one of the key strategies to enhance therapeutic efficacy.

**Figure 2:**
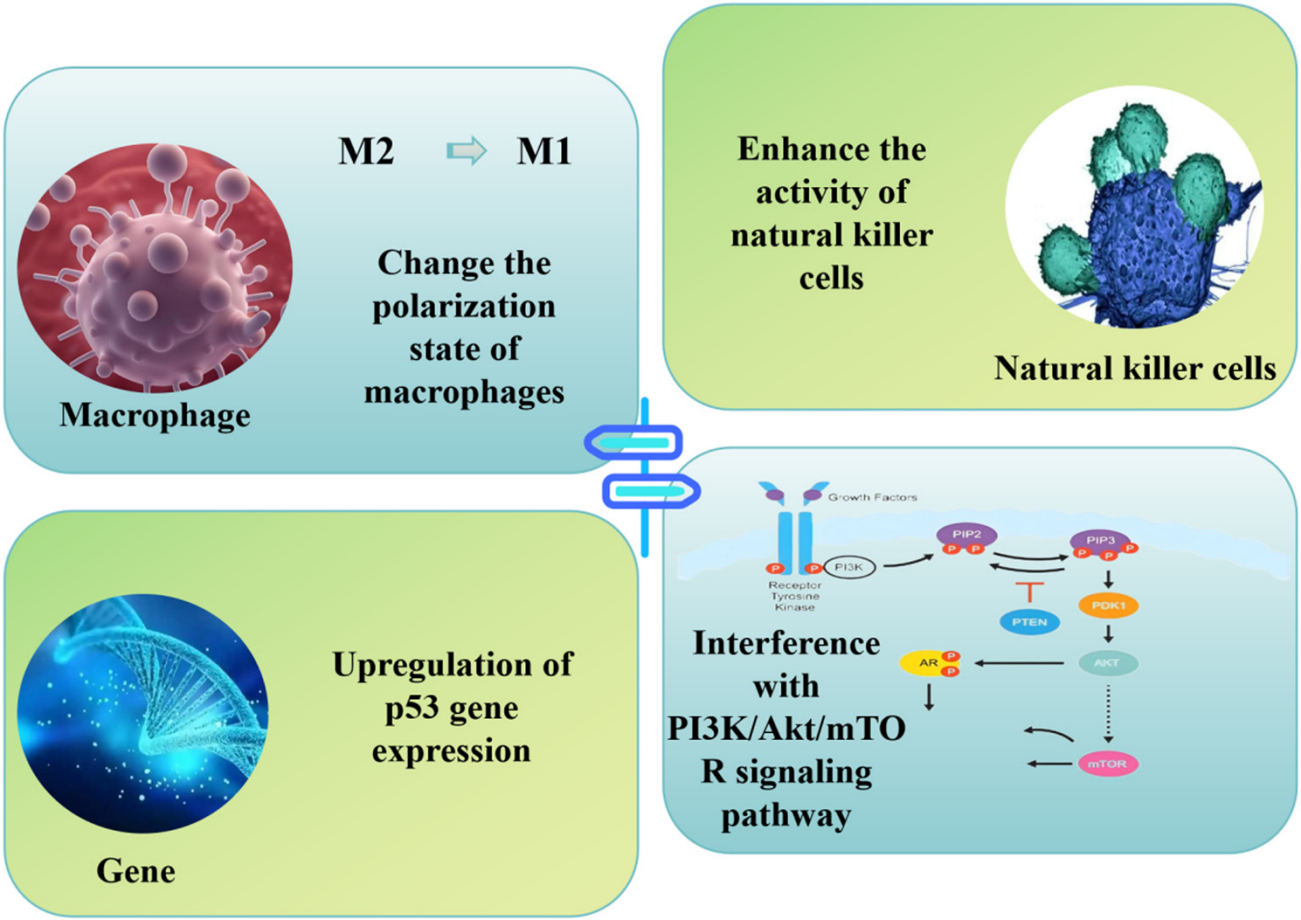
Different pathways of immune response.

The development of reliable biomarkers is crucial for guiding the selection of combination therapy with curcumin and immune checkpoint inhibitors. Existing studies have shown that biomarkers such as PD-L1 expression levels, tumor mutation burden, and microsatellite instability can predict the efficacy of immune checkpoint inhibitors. Future research should focus on identifying new biomarkers, particularly those that can reflect the mechanisms of action of curcumin, such as NF-κB activity and Nrf2 expression levels. These biomarkers can not only help identify patients who are most likely to benefit from combination therapy but also provide a basis for personalized treatment. Figure 2 illustrates the various pathways involved in immune responses.

## 4 Effects of curcumin on immune cell function

Curcumin has the capacity to modulate the activity and functionality of immune cells, including macrophages, T cells, B cells, and NK cells. In BC patients, curcumin can regulate the activity and function of tumor-infiltrating lymphocytes, thereby enhancing anti-tumor immune responses. Monocytes and macrophages constitute pivotal cells within the innate immune system, engaging in defense against invading pathogens, phagocytosis of dead cells, presentation of antigens via major histocompatibility complex class I and II molecules, and production of various pro-inflammatory cytokines and chemokines (such as interleukin [IL]-1β, IL-6, and tumor necrosis factor [TNF]-α). Upon sensing inflammatory cues, macrophages initially adopt an M1 phenotype, releasing pro-inflammatory cytokines in response. When the M1 phenotype fails to counteract stimuli and becomes extensively accumulated, it can lead to tissue damage. At this point, M2 macrophages, in contrast, secrete abundant anti-inflammatory factors, inhibiting inflammation and restoring tissue homeostasis. Tumor-associated macrophages (TAMs) polarize towards an M2 phenotype within the TME, characterized by elevated expression and release of IL-10 and transforming growth factor (TGF)-β, along with reduced IL-12. Signal transducer and activator of transcription 3 (STAT3) is a key intracellular signaling molecule involved in regulating various physiological processes, including cell proliferation, survival, apoptosis, and immune responses. In cancer, particularly in the TME, aberrant activation of STAT3 is widely recognized as being associated with the promotion of tumor progression. Under normal conditions, macrophages can be classified into two main phenotypes: M1 (classically activated) and M2 (alternatively activated). M1 macrophages promote anti-tumor immune responses by secreting pro-inflammatory cytokines, while M2 macrophages support tissue repair and suppress inflammation by secreting anti-inflammatory cytokines. However, in the TME, persistent activation of STAT3 leads to the polarization of macrophages toward the M2 phenotype, thereby promoting tumor growth and metastasis. STAT3 activation also helps tumor cells evade host immune surveillance by upregulating the expression of immune checkpoint molecules, such as PD-L1. Additionally, STAT3 is involved in regulating tumor-associated angiogenesis and epithelial–mesenchymal transition, both of which are key steps in tumor metastasis. Curcumin, as a natural compound, has been shown to effectively inhibit STAT3 activity, thereby reversing its detrimental effects on the TME. Studies have shown that curcumin can reduce the number of M2 macrophages and increase the proportion of M1 macrophages by inhibiting the STAT3 signaling pathway, thereby restoring anti-tumor immune responses [19]. In addition to affecting immune cells, curcumin can directly target tumor cells by inhibiting STAT3-mediated proliferation and survival signals, thus slowing down tumor growth. When combined with existing cancer immunotherapies, such as immune checkpoint inhibitors, curcumin may further enhance the efficacy of these therapies by modulating immune cell function in the TME and strengthening the overall anti-tumor effect. A study using the Caco-2 cell line observed that curcumin treatment significantly reduced the expression of IL-4-induced M2 macrophage markers and decreased the secretion of anti-inflammatory cytokines, suggesting that curcumin has the potential to reverse M2 polarization [20]. Another animal model study showed that mice treated with curcumin exhibited lower tumor burden and a higher M1/M2 ratio, accompanied by a stronger anti-tumor immune response [21]. In summary, curcumin, by regulating the STAT3 signaling pathway, not only inhibits tumor growth but also enhances the body’s anti-tumor immune response by remodeling the immune cell composition in the TME. These findings provide strong scientific evidence for the potential of curcumin as an adjunctive anti-cancer therapy.

## 5 Effects of curcumin on cytokine regulation

**Figure 3:**
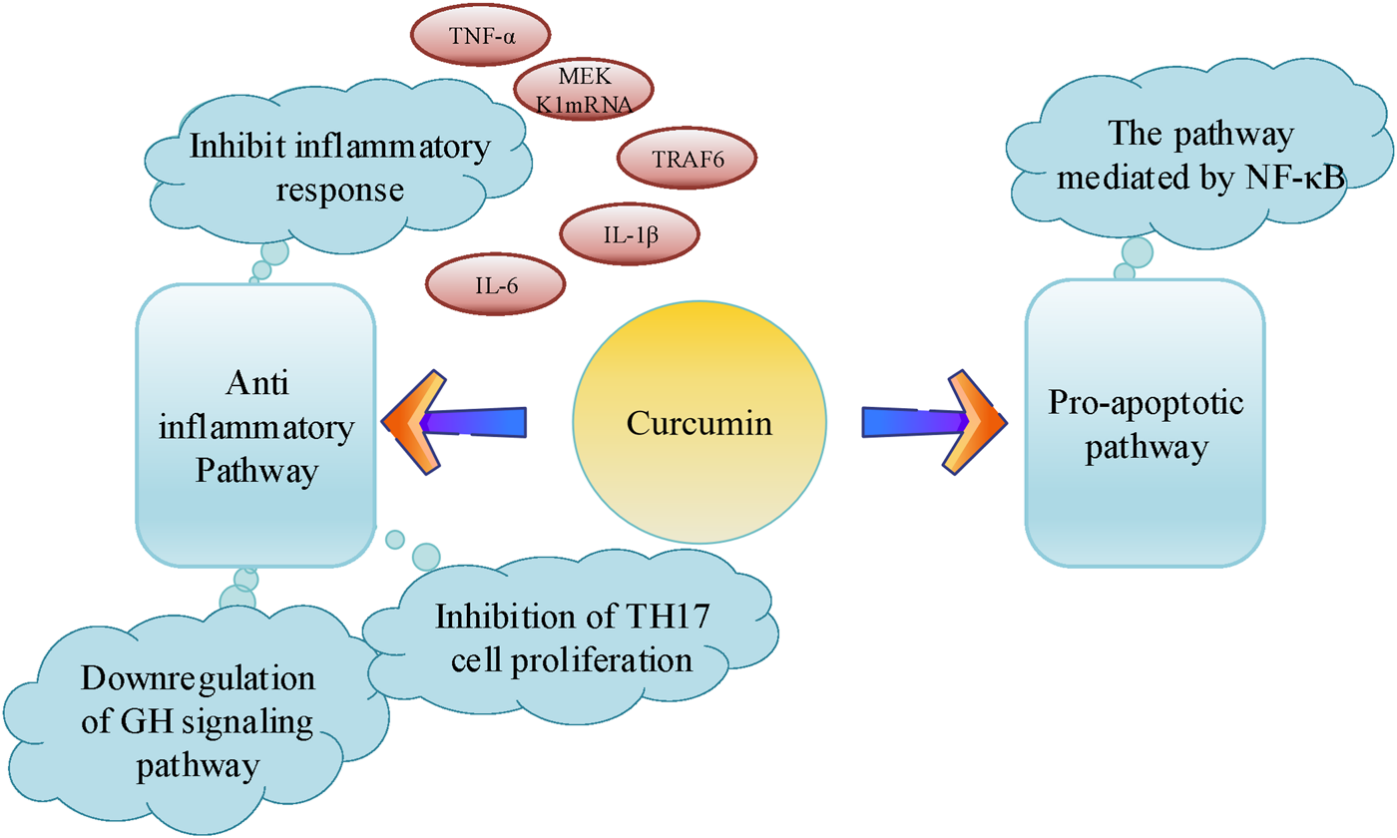
Multiple pathways of curcumin in regulating cytokines and immune responses.

Curcumin may influence the production and release of cytokines in BC cells and their surrounding microenvironment. It can modulate the balance of cytokines such as interferon-gamma (IFN-γ) and IL-2, which play a crucial role in regulating immune responses and anti-tumor effects. Dourado et al. [22] demonstrated that Th17 cells play a crucial role in promoting immune responses against extracellular pathogens by recruiting neutrophils and inducing inflammation. These cells produce inflammatory cytokines, such as TNF-α, IL-21, IL-17A, IL-23, IL-17F, IL-22, and IL-26. Curcumin has been shown to significantly inhibit the proliferation of Th17 cells and reduce the production of inflammatory cytokines, including TNF-α, IL-22, and IL-17 [23]. Figure 3 illustrates the multiple pathways through which curcumin regulates cytokines and immune responses. The pathways mainly include anti-inflammatory and pro-apoptotic pathways. Curcumin can inhibit the inflammatory response, specifically by suppressing the expression of cytokines such as TNF-α, IL-6, IL-1β, and TNF receptor-associated factor 6 (TRAF6). Additionally, curcumin can downregulate the growth hormone signaling pathway and inhibit Th17 cell proliferation, further highlighting its regulatory role in these aspects. The pro-apoptotic pathway involves the activation of nuclear factor kappa-light-chain-enhancer of activated B cells (NF-κB)-mediated signaling pathways, which promote tumor cell apoptosis. Overall, curcumin influences the production and release of cytokines through various pathways, thereby regulating immune responses and promoting tumor cell apoptosis through complex mechanisms.

## 6 Regulation of the TME by curcumin

The TME refers to the intricate milieu surrounding tumor cells, comprising a complex interplay of cellular and non-cellular components, including cells, extracellular matrix, vascular systems, immune cells, and chemical factors. Curcumin can modulate immune cells, cytokines, and signaling molecules within the TME, thereby influencing the immune response in BC. It can attenuate inflammatory responses and tumor-associated cytokine production, while enhancing immune cell functionality and activity, ultimately inhibiting tumor growth and metastasis. Research indicated that curcumin can augment the quantities of cluster of differentiation 4-positive (CD4+) T cells, cluster of differentiation 8-positive (CD8+) T cells, and IFN-γ within the TME, thereby enhancing their immune cytotoxic capabilities against tumor cells, while concurrently reducing cluster of differentiation 25 (CD25), regulatory T cells, and inflammatory cytokine levels, bolstering immune responsiveness and restoring immune surveillance capacity [24]. Cancer-associated fibroblasts (CAFs) secrete growth factors such as fibroblast growth factor, TGF-β, and vascular endothelial growth factor (VEGF), which promote tumor cell proliferation, angiogenesis, invasion, and metastasis. Studies suggest that curcumin can specifically target CAFs, thereby diminishing the invasiveness of pancreatic cancer cells [25]. Studies showed that curcumin can induce the polarization of TAMs from the pro-tumor M2 phenotype to the anti-tumor M1 phenotype. Curcumin promotes macrophage polarization toward the M1 phenotype by activating the AMP-activated protein kinase (AMPK) signaling pathway, which reduces the proliferation and migration of BC cells [26].

## 7 Effects of curcumin on tumor angiogenesis

Curcumin can impede the process of angiogenesis, thereby reducing the nutrient supply and growth of BC cells. It can disrupt tumor-associated angiogenic pathways, such as inhibiting the production of VEGF and suppressing signaling cascades involved in angiogenesis. VEGF is a glycoprotein that stimulates vascular growth, acting on receptors located on endothelial cell surfaces, such as VEGF receptor 1 (VEGFR-1) and VEGF receptor 2 (VEGFR-2), to induce endothelial cell proliferation and migration, ultimately leading to neovascularization. Overexpression of VEGF results in the formation of aberrant blood vessels, supplying ample nutrients to tumors and fostering tumor growth and metastasis. Research indicated that curcumin can inhibit VEGF expression by impeding proteases involved in the degradation of vascular basement membrane and extracellular matrix, thereby preventing the generation and dissemination of new tumor-associated blood vessels [27]. Furthermore, studies demonstrated that the combined use of curcumin and Astragalus polysaccharides can induce normalization and sparse remodeling of tumor vasculature morphology [28].

## 8 Research and adoption of curcumin as a therapeutic approach for BC

Conventional therapeutic approaches for BC include surgical intervention, radiotherapy, and chemotherapy. In non-conventional scenarios, endocrine therapy, targeted therapy, and immunotherapy are also employed. However, each of these approaches presents certain limitations in clinical practice. Curcumin, as a natural compound, exhibits remarkable potential in cancer therapy, underscoring its substantial promise as an alternative or adjunctive treatment for BC.

### 8.1 Surgical treatment

Surgery constitutes a fundamental therapeutic modality for BC management. Depending on tumor size and staging, options include breast-conserving surgery (tumor excision with preservation of surrounding breast tissue) or total mastectomy (complete removal of the breast). In the context of BC treatment, axillary lymph node dissection may be necessary preoperatively or postoperatively to determine lymph node involvement by cancer cells. This aids in staging the tumor and guiding subsequent treatment decisions. Patients with lymph node metastasis can exhibit postoperative recurrence rates exceeding 70%, while those without lymph node involvement may still experience recurrence rates surpassing 20%.

When combined with surgical treatment, the synergistic effects of curcumin are as follows: (i) anti-inflammatory and anti-edema effects: the anti-inflammatory and antioxidant properties of curcumin can alleviate postoperative inflammation and swelling in patients. By reducing the inflammatory response, curcumin may alleviate postoperative discomfort and expedite wound healing. (ii) Reduction of surgical site recurrence risk: curcumin exhibits anti-tumor activity by modulating the NF-κB and PI3K/AKT mitogen-activated protein kinase pathways, thereby inhibiting tumor cell proliferation and metastatic potential. Following surgical intervention, adjunctive use of curcumin might contribute to reducing the risk of recurrence from residual tumor cells. Research indicates that curcumin exerts its effects on MDA-MB-231 BC cells by inducing AMPK activation, leading to autophagy induction, and suppressing AKT levels, thus inhibiting tumor cell proliferation and migration [29]. The cytotoxic effects of curcumin on BC cells have been shown to depend on their PI3K–AKT signaling status. In BC cells with active PI3K–AKT pathway, curcumin can inhibit the excessive activation of this pathway, thereby suppressing cell proliferation and promoting apoptosis [30]. Conversely, the cytotoxic effects of curcumin may be relatively limited in BC cells with an inhibited PI3K–AKT pathway. Compared to MDA-MB-231 cells, curcumin treatment of MCF-7 cells requires higher doses and longer treatment durations to maximize AKT phosphorylation and induce cytotoxicity.

In clinical applications, the low bioavailability of curcumin presents a major challenge. To address this issue, researchers have developed various formulations, such as nanoparticles, liposomal encapsulation, and phospholipid complexes, to enhance the stability and absorption efficiency of curcumin [31]. In human studies, oral capsules or tablets are commonly used, with doses ranging from several hundred milligrams to several thousand milligrams per day, depending on individual variations and therapeutic objectives. To ensure safety and efficacy, personalized dosing strategies are essential and should be adjusted based on the patient’s physiological condition and disease progression.

In addition to *in vitro* studies, several *in vivo* studies have demonstrated the potential value of curcumin in BC treatment. For instance, in mouse models, curcumin has been shown to significantly inhibit tumor growth and exhibit enhanced anti-tumor effects when combined with chemotherapeutic agents such as paclitaxel [32]. Another study found that curcumin can enhance anti-tumor immune responses by modulating the immune microenvironment, thereby reducing the risk of postoperative recurrence [33]. These *in vivo* studies provide strong support for the potential application of curcumin in human BC treatment and highlight its substantial potential for translation into clinical practice.

Although most current research on curcumin remains in the experimental stage, with the advancement of more high-quality clinical trials, particularly those evaluating curcumin as an adjunctive therapy, we expect to gain a better understanding of its role in the comprehensive treatment of BC. Future studies should continue to explore the optimal administration protocols for curcumin, assess its long-term safety and efficacy, and investigate the best combinatory approaches with existing treatments.

### 8.2 Radiation therapy

Radiation therapy utilizes high-energy X-rays or other forms of radiation to irradiate the breast or lymph node regions, targeting residual tumor cells to reduce tumor recurrence and enhance local control rates. Radiotherapy is often employed following breast-conserving surgery to mitigate the risk of local recurrence. Radiotherapy may be administered over several weeks or months following surgery, typically involving multiple treatment sessions. For stage I and II BC patients, a standard regimen of 3–6 weeks of radiotherapy, administered five times weekly, is commonly applied for disease control. In contrast, stage III and IV BC patients may receive approximately three weeks of preoperative radiotherapy, followed by surgical intervention and subsequent comprehensive treatment, including radiotherapy and chemotherapy, with a total of no more than 40 radiation sessions. When combined with radiation therapy, curcumin can act as a radioprotectant for normal cells in BC patients. Research indicates that the adjunct use of curcumin can effectively enhance the efficacy of radiation therapy while reducing radiation-induced damage to normal tissues [34].

Curcumin has been shown to inhibit the activation of the NF-κB pathway, downregulate the expression of pro-inflammatory cytokines, and reduce the activity of matrix metalloproteinases, thereby decreasing the anti-apoptotic capacity of tumor cells and making them more sensitive to radiation. Studies demonstrated that curcumin-treated MDA-MB-231 cells exhibit increased sensitivity to radiation therapy, which is associated with the inhibition of the PI3K/AKT signaling pathway [35]. Promoting tumor cell apoptosis and autophagy: Curcumin can induce autophagy by activating the AMPK signaling pathway, suppress AKT levels, and affect MDA-MB-231 BC cells, thereby inhibiting tumor cell proliferation and migration. This effect helps enhance the selective cytotoxicity of radiotherapy on tumor cells, reducing the number of residual tumor cells.

### 8.3 Chemotherapy

Curcumin, a natural polyphenolic compound extracted from turmeric, has shown significant potential in the treatment of BC in recent years, particularly as an adjunctive therapy to chemotherapy. Although traditional chemotherapeutic agents such as anthracyclines, taxanes, and fluorouracil exhibit notable efficacy in controlling and eliminating tumors, their non-specific effects lead to damage to normal cells, causing a range of side effects, and some patients develop resistance to these drugs. Curcumin enhances the effectiveness of chemotherapy through various mechanisms while alleviating its side effects, making it a promising adjunctive therapeutic strategy.

Curcumin can enhance the efficacy of chemotherapeutic agents while reducing their toxic side effects. Several studies demonstrated that when used in combination with chemotherapy drugs, curcumin significantly improves treatment outcomes. For example, in *in vitro* experiments, curcumin combined with doxorubicin exhibited a synergistic effect on BC cell lines, not only enhancing the cytotoxicity of doxorubicin but also reducing the oxidative stress and DNA damage induced by doxorubicin [13]. Similarly, in animal models, the combination of curcumin and paclitaxel significantly increased tumor suppression rates and prolonged the survival of mice [36]. These findings suggest that curcumin not only enhances the anticancer effects of chemotherapy drugs but also minimizes their damage to normal tissues.

Curcumin can also overcome chemotherapy resistance in cancer cells. Multidrug resistance (MDR) is one of the major causes of chemotherapy failure, primarily mediated by P-glycoprotein (P-gp), which pumps chemotherapy drugs out of cells, reducing intracellular drug concentrations. Studies have shown that curcumin can reverse chemotherapy resistance by inhibiting the expression or function of P-gp. Curcumin downregulates P-gp expression, increasing the accumulation of doxorubicin in drug-resistant BC cells, thereby restoring their sensitivity to the drug [37]. Furthermore, curcumin can regulate other resistance-related genes and proteins, such as Bcl-2, BCL2-associated X protein (Bax), and caspase-3, to promote cancer cell apoptosis, further enhancing the effectiveness of chemotherapy drugs [8,9].

### 8.4 Endocrine therapy

Curcumin has shown significant potential in overcoming resistance to endocrine therapy. Endocrine therapy resistance is one of the major challenges faced by HR + BC patients and is typically caused by various mechanisms, such as ER mutations, activation of bypass signaling pathways, and epigenetic alterations. Through its multitargeted mechanisms, curcumin can reverse these resistance mechanisms. In addition to its direct antitumor effects, curcumin possesses immunoregulatory functions that can exert a synergistic effect in endocrine therapy. Immunosuppressive cells present in the BC microenvironment, such as TAM and myeloid-derived suppressor cells, can weaken the body’s antitumor immune response. Curcumin enhances this response by inhibiting the STAT3 signaling pathway, altering the phenotype of macrophages from the M2 type to the more antitumor-active M1 type [38]. Moreover, curcumin can upregulate the functions of NK cells and T cells, enhancing the body’s ability to recognize and kill tumor cells, thereby further improving the effectiveness of endocrine therapy.

When combined with traditional endocrine therapy drugs, curcumin can significantly enhance therapeutic efficacy while reducing side effects. Multiple studies have demonstrated that curcumin when used in combination with tamoxifen, can enhance its antitumor activity. One study showed that curcumin also exhibited a synergistic effect when combined with aromatase inhibitors, significantly increasing tumor suppression rates and extending the survival of mice [39].

### 8.5 Targeted therapy

Targeted therapy involves using drugs to intervene in specific targets within BC cells. For example, HER2-positive BC can be treated with HER2 antibody drugs (such as trastuzumab) or HER2 receptor tyrosine kinase inhibitors (such as lapatinib). Most currently used targeted therapies affect healthy cells throughout the body. In contrast, curcumin, a natural antioxidant, has low bioavailability. To enhance its absorption efficiency, mesoporous silica nanoparticles (MSNPs) combined with hyaluronic acid (HA) have been employed as a drug delivery system. The research noted that compared to free curcumin, the synthesized targeted drug reduces tumor volume in mice by inducing reactive oxygen species (ROS), cell cycle arrest, and regulating the NF-κB and Bax-mediated apoptotic pathways [40]. MSNPs conjugated with HA have been utilized as drug delivery systems to significantly enhance the bioavailability and targeting ability of curcumin. Studies showed that compared to free curcumin, the synthesized targeted drug reduces tumor volume in mice by inducing ROS, cell cycle arrest, and modulating NF-κB and Bax-mediated apoptosis pathways [41]. Furthermore, this nanoparticle delivery system enhances therapeutic efficacy by prolonging blood circulation time and increasing accumulation in tumor tissues. Liposomes, small vesicles composed of phospholipid bilayers, can encapsulate curcumin within their interior or embed it in the membrane, thereby protecting it from degradation and promoting cellular uptake. Research showed that curcumin encapsulated in liposomes exhibits greater stability and improved bioavailability *in vivo* while reducing toxicity to normal tissues [42]. Additionally, liposomes can achieve active targeting through surface modification, further improving the selectivity and efficacy of the drug. Polymeric nanoparticles represent another effective drug delivery platform, capable of maintaining drug concentrations by controlling release rates, thus enhancing therapeutic outcomes. Studies have shown that curcumin delivered by PLGA nanoparticles not only improves bioavailability but also enhances selective cytotoxicity against tumor cells [43]. Additionally, PLGA nanoparticles can be surface-functionalized to incorporate targeting ligands, such as folic acid or antibody fragments, enhancing their recognition and binding ability to specific tumor cells. Hydrogels, formed by crosslinking hydrophilic polymers into a three-dimensional network, provide a sustained-release drug delivery environment at the local site. Curcumin-loaded hydrogels can achieve prolonged drug release at the tumor site while maintaining a high local concentration, thereby improving therapeutic efficacy and reducing side effects. Furthermore, hydrogels can be combined with other therapeutic modalities, such as chemotherapy or immunotherapy, to create integrated treatment strategies.

### 8.6 Immunotherapy

Immunotherapy involves utilizing the activation or enhancement of the patient’s own immune system to combat BC. In addition to leveraging its inherent anti-inflammatory and antioxidant properties for anticancer effects, curcumin can exert potent antiproliferative potential against BC cells by inhibiting the cell cycle and inducing apoptosis. Enhancer of zeste homolog 2 (EZH2) is a frequently upregulated epigenetic factor in human cancers, while deleted in liver cancer 1 (DLC1) is an anticancer gene that is often expressed at low levels or not expressed in many malignancies. EZH2 exhibits high expression levels in BC patients, whereas DLC1 is lowly expressed. Research has demonstrated that curcumin restores DLC1 expression by inhibiting EZH2, concurrently suppressing migration, invasion, and proliferation of MDA-MB-231 cells (MDA-MB-231), promoting apoptosis, and arresting the cell cycle [44]. Investigation into the impact of dendrimer-based nano-curcumin on p53-mutant BC cells revealed significant modulation of P-glycoprotein function, potentially enhancing the cellular retention of curcumin. The disruption of P-glycoprotein activity by dendrimer nanocurcumin suggests its ability to attenuate drug resistance in p53-mutant cancer cells [45]. P-glycoprotein (P-gp) is a member of the ATP-binding cassette (ABC) transporter family encoded by the ATP-binding cassette sub-family B member 1 (ABCB1) gene. It is located on the cell membrane and functions to pump a variety of chemotherapeutic drugs out of the cell, thereby reducing intracellular drug concentrations and leading to MDR. MDR is one of the major causes of chemotherapy failure, enabling tumor cells to develop resistance to multiple structurally and functionally distinct drugs. P-gp not only affects the efficacy of chemotherapy but may also impact the effectiveness of immunotherapy, as it can pump drugs such as immune checkpoint inhibitors out of the cell, thereby reducing their therapeutic efficacy. The expression level of the ABCB1 gene is closely associated with MDR. High expression of ABCB1 often predicts poor treatment response and survival outcomes. Clinically, measuring ABCB1 expression levels can help predict patients’ treatment responses and guide the selection of personalized treatment strategies. For example, in certain cases, the use of ABCB1 inhibitors in combination with chemotherapy or immunotherapy can enhance therapeutic efficacy and overcome drug resistance. Furthermore, polymorphisms in the ABCB1 gene may also influence drug metabolism and therapeutic efficacy, suggesting that these genetic factors should be considered when developing treatment plans. Studies showed that curcumin can modulate P-gp function through multiple mechanisms, thereby reversing MDR [46]. First, curcumin can directly inhibit the expression and activity of P-gp, leading to increased accumulation of chemotherapeutic drugs within cells and enhancing treatment sensitivity. Second, curcumin can further improve chemotherapy efficacy by downregulating the expression of the ABCB1 gene. Research has also demonstrated that curcumin can enhance anti-tumor effects through other mechanisms, such as inhibiting the NF-κB pathway and the PI3K/AKT signaling pathway [18].

When evaluating curcumin as a treatment for BC, it is important to acknowledge the limitations of existing preclinical study designs, including small sample sizes and physiological differences between animal models and humans, which may limit the generalizability of the results. Conflicting findings across different studies may be attributed to variations in dosage, formulation, and experimental conditions. Furthermore, certain delivery strategies or combination therapies have failed to achieve the expected outcomes, such as instability of specific nanoparticle formulations or adverse interactions with chemotherapy drugs, issues that require further investigation and optimization. Methodological challenges also include complex pharmacokinetic properties and bioavailability issues, both of which are key factors influencing curcumin’s efficacy. Although many preclinical studies have demonstrated curcumin’s significant potential, clinical trials have often failed to fully replicate these positive results, highlighting a gap between preclinical promise and actual clinical outcomes. This gap underscores the need for caution when interpreting and applying preclinical data, and emphasizes the importance of further optimizing curcumin delivery systems and therapeutic strategies. By thoroughly examining these aspects, future research can provide more valuable direction and guidance, advancing the practical application of curcumin in BC treatment.

When exploring the effects of curcumin on immune responses in BC, it is important to consider several clinical aspects. First, dosage recommendations based on existing evidence should be approached with caution, and further research is needed to clarify its efficacy and safety. Second, clinical trials related to curcumin and BC treatment are ongoing, investigating the potential effects under specific therapeutic regimens. Additionally, understanding how curcumin may synergize with standard therapies is a key area of research, as it could help optimize treatment strategies. Patient stratification is crucial for identifying which populations are most likely to benefit from curcumin supplementation while considering the quality of life factors is an essential part of evaluating any treatment’s impact. In conclusion, although preliminary studies suggest that curcumin may have the potential to modulate immune responses, all medical and health information should be considered a reference only, with specific treatment plans developed under the guidance of healthcare professionals to ensure safety and effectiveness.

## 9 Conclusion

Curcumin, a natural compound, exhibits broad-spectrum anti-cancer activity and shows potential immune-modulatory effects. It influences immune responses through various mechanisms, including the regulation of immune cell functions, alteration of the TME, and modulation of immune cell activity. Its unique immune-regulatory properties enable curcumin to enhance the effects of immune cells on cancer cells, significantly inhibiting cancer cell proliferation, inducing autophagy, regulating the cell cycle, and promoting apoptosis. The low water solubility and rapid metabolism of curcumin limit its ability to maintain effective concentrations *in vivo*. Although novel delivery systems, such as nanotechnology and liposomal encapsulation, offer potential solutions, further validation is required before clinical application. Future research should focus on developing more effective delivery systems to overcome bioavailability issues, formulating personalized dosing regimens based on individual differences, and gaining a deeper understanding of the specific mechanisms of action of curcumin, particularly in terms of how it selectively affects tumor cells without damaging normal tissues. Additionally, larger-scale and long-term studies are needed to ensure its safety and efficacy as a therapeutic option. Furthermore, exploring the interactions between curcumin and other existing anti-cancer drugs or immune checkpoint inhibitors to identify optimal combination strategies could enhance therapeutic outcomes.
